# Fault-Adaptive Autonomy in Systems with Learning-Enabled Components

**DOI:** 10.3390/s21186089

**Published:** 2021-09-11

**Authors:** Daniel Stojcsics, Dimitrios Boursinos, Nagabhushan Mahadevan, Xenofon Koutsoukos, Gabor Karsai

**Affiliations:** Institute for Software Integrated Systems, Vanderbilt University, Nashville, TN 37212, USA; dimitrios.boursinos@vanderbilt.edu (D.B.); nag.mahadevan@vanderbilt.edu (N.M.); xenofon.koutsoukos@vanderbilt.edu (X.K.); gabor.karsai@vanderbilt.edu (G.K.)

**Keywords:** assured autonomy, behavior trees, learning-enabled component, assurance monitor, fault detection and isolation, BlueROV, ROS

## Abstract

Autonomous Cyber-Physical Systems (CPS) must be robust against potential failure modes, including physical degradations and software issues, and are required to self-manage contingency actions for these failures. Physical degradations often have a significant impact on the vehicle dynamics causing irregular behavior that can jeopardize system safety and mission objectives. The paper presents a novel Behavior Tree-based autonomy architecture that includes a Fault Detection and Isolation Learning-Enabled Component (FDI LEC) with an Assurance Monitor (AM) designed based on Inductive Conformal Prediction (ICP) techniques. The architecture implements real-time contingency-management functions using fault detection, isolation and reconfiguration subsystems. To improve scalability and reduce the false-positive rate of the FDI LEC, the decision-making logic provides adjustable thresholds for the desired fault coverage and acceptable risk. The paper presents the system architecture with the integrated FDI LEC, as well as the data collection and training approach for the LEC and the AM. Lastly, we demonstrate the effectiveness of the proposed architecture using a simulated autonomous underwater vehicle (AUV) based on the BlueROV2 platform.

## 1. Introduction

In recent years, with the rapid development of GPU-accelerated embedded computers, there is a steadily growing number of Learning-Enabled Cyber-Physical Systems [1] among autonomous vehicles in the air, ground, sea, and undersea domains. ‘Learning-Enabled’ means that certain functions are implemented using machine-learning techniques—usually deep learning—resulting in models that implement these ‘Learning-Enabled Components’ (LECs) [2]. The machine-learning methods used in the design of LECs rely on training data collected via system operations or simulation. For training and evaluation, multiple simulation runs are necessary so that the training data sufficiently covers the spectrum of operational situations that the autonomous vehicle will encounter. Such autonomous vehicles are often operated in hazardous environments with a high degree of uncertainty, where the safety of the vehicle and mission success depend highly on the performance of the LECs onboard. ‘Assured Autonomy’ is a paradigm for improving the performance of LECs, where safeguards are integrated into the system’s design to provide guarantees (‘assurances’) for safe and robust behavior, even when a component (possibly an LEC trained with incomplete data) does not work correctly.

Faults and degradations, e.g., sensor failures, software malfunctions, actuators degradations, etc., can happen anytime and anywhere in a system. This paper focuses on faults and degradations in the actuators that can considerably affect the system behavior. We propose a system architecture where a subsystem—the autonomy manager—monitors the vehicle hardware’s and software for anomalies and takes corrective actions as needed. The autonomy manager includes an LEC for fault detection and isolation (FDI). The manager also includes an additional component called the ‘assurance monitor’ that supervises the LEC and determines the confidence that the generated output is correct. The results of these two components are combined into a single decision for initiating a change in the controllers that would mitigate the actuator fault.

We have implemented this system architecture using Gazebo-based simulation and Robot Operating System (ROS) with Python nodes. The autonomy manager is using a Python-based Behavior Tree (BT), and the autonomous underwater vehicle (AUV) is based on the 6 thruster-driven BlueROV2 platform. Details of the implementation can be found in Section 4

The contributions of this paper are:

- The novel system architecture that ensures that the mission execution is robust to faults and anomalies,
- The use of an assurance monitor that complements the FDI LEC predictions with credibility and confidence metrics,
- The design of an assurance evaluator that decides whether a particular classification can be trusted or not; this decision-making process is based on requirements related to the acceptable risk of each decision as well as the desired frequency of accepted classifications.
- The evaluation of the fault-adaptive system using an AUV example in ROS/Gazebo-based simulations with more than 400 executions for various hazardous/faulty environments.

The paper is organized as follows. In Section 2 we provide a brief overview of FDI for autonomous vehicles, how behavior trees (BT) are used as higher-level autonomy controllers, and how LECs can be used in autonomous systems. In Section 3 we present our high-level architecture for an autonomous vehicle based on the BlueROV2 system, with fault-adaptive autonomy in focus, as well as the metrics for evaluation. Section 4 presents details of: (1) the chosen autonomous vehicle (AV), including implementation details focusing on the autonomy manager and BT-based mission execution, and (2) the LEC, augmented with assurance monitoring technology. Finally, in Section 5 and Section 6 the paper concludes by summarizing the results and makes recommendations for further research.

## 2. Background

### 2.1. Autonomous Vehicles

Fault detection, isolation (FDI) is widely used technology [3] in many disciplines, including aerospace engineering and automotive systems. In such systems, there is a high degree of redundancy to achieve robust fault detection and appropriate control reconfiguration. Drive-by-wire and fly-by-wire systems are examples for proven solutions. In general, there is an open loop plant with actuator(s), plant dynamics and sensor(s) to model the system and the fault. These systems usually include redundant actuators and sensors to achieve robust operation. Faults can occur within an actuator, sensor or component (software or hardware such as Extended Kalman Filter or power module) fault, and the system must detect and react to these accordingly. FDI algorithms can be designed to be robust to disturbances and noise and can be grouped to a few basic approaches, all of which are based on residual generation which is sensitive to faults but robust to noise. With the fault isolation comes the decision-making based on statistical tests of residuals. Reconfiguration is a common contingency action in response to a fault and usually involves some sort of controller change in the system to maintain satisfactory operation.

Existing work on FDI is very broad and there are techniques which have been applied to autonomous systems. An FDI method in Electric Ship Power Systems (SPS) for the next-generation US Navy fleets is presented in [4]. As a highly critical system, an SPS operates with overlapping fault-tolerance, reliability, security to maintain survivability. This is not only important in military systems but also for unmanned autonomous vehicles such as Unmanned Aerial Vehicles (UAVs) and Unmanned Underwater Vehicles (UUVs). An FDI method to guarantee system safety and reliability for UAVs with sensor faults using state and input estimation is presented in [5]. A robust and efficient fault diagnosis system for quadrotor UAV actuator failures based on quasi linear parameter varying (qLPV) systems is presented in [6]. A robust $H_{\infty }$ observer is designed to detect partial and total degradation for actuators and tested with numerical experiments in MATLAB/Simulink. FDI with fault-tolerant control for the DJI F550 hexacopter platform and open source Pixhawk2 flight controller with APM Copter v.3.5 and custom C++ user implementation code is used in [7]. In [8], the authors present a fault-tolerant control for open-frame ROVs using control reallocation and power isolation. The presented vehicle has 8 or 12 thrusters, so the hardware layer redundancy for control is much higher than the standard BlueROVs. Multiple and different partial and total degradations were presented such as jammed, broken or lost propellers. For control reallocation, they use a fixed fault code table with thruster saturation bounds and thruster power on/off switch. A grey prediction rank particle filter (GP-RPF) fault diagnosis method for a thrust loss detection and control reallocation problem on a four-thruster drive AUV is presented in [9]. The thruster layout is different than the BlueROV2 thrusters, but the main idea is to detect the faulty thruster and reallocate thrust to balance thrust and torque loss, similar to our work. In our work, we use an LEC to obtain the degradation information, without using the AUV position and status plus the predicted status information.

### 2.2. LECs in Autonomous Vehicles

A central problem with LECs in an autonomous system is that the performance is dependent on the training data. When the neural network receives out of distribution data that differs significantly from the distribution of the training data, the precision and recall drops dramatically [10]. As autonomous vehicles become increasingly complex, it is hard to fully test and validate such systems offline. A method for verifying the safety of autonomous systems with LEC controllers with sigmoid/tanh activations is introduced in [11]. Assured runtime monitoring and planning to provide safe operation in an environment with unsafe levels of disturbance and noise is presented in [12]. A novel assume-guarantee-based verification approach for automotive and autonomous LECs is presented in [13]. The input for such neural network-based automotive systems are based on cameras along with LiDAR sensors. A trained LEC controller processes the inputs and creates control outputs for obstacle avoidance while using reachability analysis to compute the set of safe states [14]. In [15], a controller verification scheme for detecting unstable learning behaviors is presented for online neural networks. The situation is similar in the case of small Unmanned Aircraft Systems (UAS) with large numbers of such vehicles entering the airspace. These vehicles use LECs for sensing, guidance and navigation. Thus, formal verification and assurance can improve safety and ease the integration of these vehicles into the national airspace [16].

Typically, CPSs are used in applications where wide range of autonomy is required, for example, in unsafe operation conditions or uncertain and hazardous environments. To develop the software required for such systems, we are using the Assurance-based Learning-enabled Cyber-physical systems (ALC) Toolchain [17], developed at Vanderbilt University. This toolchain supports full development cycle for CPS design with LECs [18] including architectural modeling, data collection, LEC construction, verification and system-level assurance.

### 2.3. Behavior Trees for Autonomy

Behavior Trees (BT-s) were originally created for providing ‘artificial intelligence’, i.e., autonomous and/or reactive behaviors in computer games. However, in recent years BT-s appeared in high-level mission management logic in autonomous systems [19]. BT-s can be used to create high-level autonomy controllers, similar to Hierarchical Finite State Machines (HFSM). Although HFSMs are rigid structures and become less and less manageable as the complexity of the system grows with additional features, BT-s are much easier to expand and maintain. An example of a complex BT high-level autonomy for an air-to-air combat UAV is presented in [20]. In this example, the presented tree demonstrates the abilities of the BT. A BT is a directed graph with nodes and edges in a strict parent/child order. Each node can have multiple children and at most one parent. The node without a parent is the root—the top-level entity, and nodes without children are the leaves. Non-leaf nodes can be of type Selector, Sequence, Parallel and Decorator, while leaves can be Actions (Tasks) or Conditions. The state of a node can be Running, Success or Failure. With these simple building blocks, a complex high-level autonomy controller can be created with simple a construction process. BT-s have a basic execution semantics as follows: (1) Prioritized execution can be done using Selectors, where the ordering of the children nodes provides the priority order, (2) Sequential execution is done with Sequence nodes, where each previous node must either Succeed or Fail to trigger the next operation, and (3) Parallel execution of tasks (and complete sub-trees) is also supported. In such a structure, it is easy to insert additional actions, but it is more manageable than an equivalent HFSM.

BT-s also have a shared data structure called Blackboard (BB) where nodes can read and modify the variables stored there [21], similar to a set of global public variables. The BB variables can be atomic or compound data types. A BT is periodically evaluated, typically with a fix rate, e.g., 1 HZ, so every running node executes an “update” method with this rate.

## 3. System Architecture

**Figure 1:**
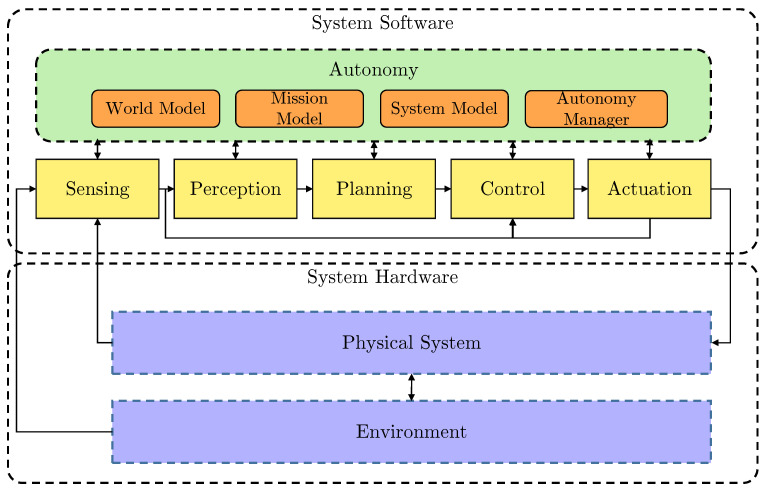
Simplified high-level system architecture.

Figure 1 shows the simplified high-level architecture of the AUV system. The example BlueROV2 system architecture used in this paper supports BT-based robust mission execution. This includes robustness to faults, anomaly detection and contingency action with decisions based on a LEC with an AM.

### 3.1. Fault-Adaptive Autonomy

The system is divided into hardware, which is the AUV itself including thrusters, actuators, CPU, etc., and software, which includes the autonomy engine and sensing-to-actuation pipeline. The autonomy engine consists of a world model which describes the AUV’s operating environment, including the occupancy grid-based pipeline map, obstacle map and other smaller maps for navigation and guidance. The mission model describes the AUV mission with the help of a mission file (what to execute) and a mission server (how and when to execute). The system model is a representation of the AUV operational state, either nominal or degraded, and any identified hazards such as low battery or obstacle detected. The autonomy manager implements the higher-level autonomy in the system, including the mission execution, and LEC-based fault-adaptive operation.

In the System Software stack, there is a complete low-level pipeline from sensing to actuation, supplying the inputs for the autonomy and the control output for the AUV. There are complex sensors, including Side Scan Sonars (SSS) for infrastructure mapping [22], Forward Looking Sonars (FLS) for obstacle avoidance [23] and altitude measurement, and basic ones such as rotation-per-minute (RPM) and motor current sensors. In the perception block, there are multiple LECs for processing the sensor data (e.g., there is a Semantic Segmentation Convolutional Neural Network (SemSeg CNN) [24] for SSS and a Long Short-Term Memory Recurrent Neural Network (LSTM RNN) [25] type for FLS). The planning part processes the filtered sensory data (LEC outputs from the perception stage) and based on the Autonomy it produces a Heading–Speed–Depth (HSD) command for the control block. The control block uses the dynamic positioning controller available from ‘UUV Simulator’ [26,27] package and makes a control output based on the HSD commands. These commands drive the AUV’s 6 thrusters in the physical layer with PWM signals using MAVROS [28] conversion and a Pixhawk as a middleware unit (implementing the command to PWM signal conversion).

**Figure 2:**
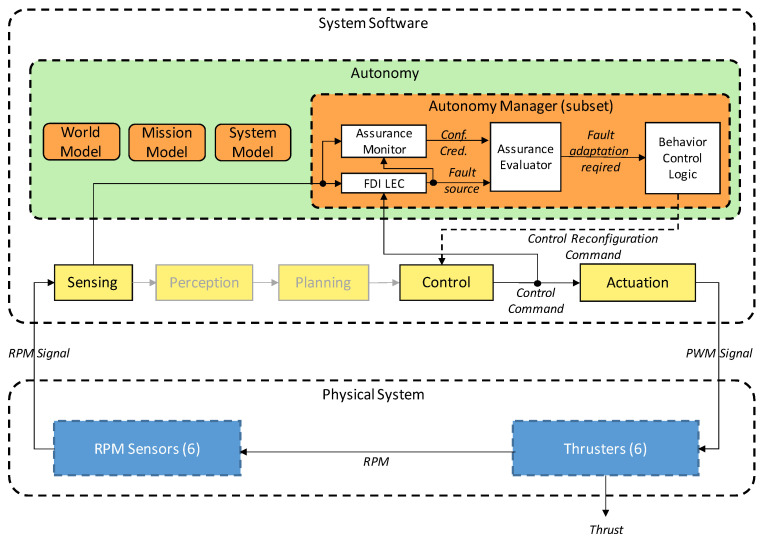
Detailed system architecture—focusing on the Autonomy Manager.

The autonomy manager the focus of this paper (Figure 2). It contains an LEC-based fault detection and isolation (FDI) module along with an AM. If the LEC with the AM detects a thruster failure and the Assurance Evaluator verifies this fault, then the information is passed to the BT control logic. Under autonomous operation, the FDI subsystem is always running. It receives thruster RPM signals from the sensors and control commands from the Control node. If the LEC and AM are indicating that the AUV is in nominal state, and the Assurance Evaluator confirms this output, there is no need for reconfiguration.

If the LEC detects a degradation (*Fault source*), the AM calculates the *Credibility* and *Confidence* metrics for that output. Using these values, the Assurance Evaluator marks the output as reliable with a *Fault Adaptation Required* signal, and fault adaptation is initiated. The LEC output class determines the degraded thruster (Actuator) and the approximate degradation level. Based on this information, the Control node can perform a control reallocation using a Thruster Allocation Matrix (TAM) which allows the AUV to continue the mission. The details of the LEC and AM can be found in Section 4.3.1.

In our simulation environment, the Physical System (sensors, thrusters, etc.) is modeled using Gazebo 9 [29]. Real world operation uses a Linux-based embedded computer (Raspberry Pi3b or Nvidia Jetson TX1/2) with Ubuntu 16.04 LTS and ROS Kinetic with MAVROS package (ROS to Mavlink conversion) and a compatible Pixhawk1 autopilot with ArduSub 4.0+ firmware [30].

For the data generation process, we capture all the messages exchanged between ROS nodes in ROS Bagfiles with a standard ROS internal service. The data collection is provided by the ROS simulation environment, and we rely on the internal ROSbag mechanism [31]. We use the messages in the ROS Bagfiles as the source of training data for LEC Development workflow provided by ALC-Toolchain. The training itself is a design-time process. In the runtime system there is no explicit data collection for training.

### 3.2. Evaluation Metrics

To evaluate the performance of the proposed architecture, multiple mission simulations must be executed with obstacles and obstacle avoidance. The metrics available and used for evaluation are as follows:

- FDI LEC recall and accuracy (Ground truth vs. LEC output)
- FDI LEC recall and accuracy with AM and assurance evaluator (Ground truth vs. LEC + AM + AE output)
- Mission execution time (s)
- Average cross-track error during mission (m)

Recall is defined as *True Positives/(True Positives + False Negatives)*, accuracy is defined as *(True Positives + True Negatives)/All samples*.

For real-time operation, the goal is an overall high recall and accuracy value—accepting all correct LEC outputs while rejecting incorrect ones. Using these metrics, we can compare the performance of our method compared to the raw LEC output. Mission execution time and average cross-track error are calculated in real time, during the mission. Using these metrics, we can compare the effect of the control reallocation compared to the degraded operation without FDI.

## 4. Approach

### 4.1. Vehicle Details

In this paper, we use the ‘UUV Simulator’ which is a Gazebo-based ROS package for Unmanned Underwater Vehicles (UUVs). Both AUV-s and Remotely Operated Underwater Vehicles (ROV) can be simulated with this package. The simulator supports the development of two different kinds of underwater vehicles. The first type is thruster only ROVs, operated in a small region using a tethering cable, such as BlueROV (http://bluerobotics.com/store/rov/bluerov2), RexROV and Desistek SAGA. These precise, thruster-controlled robots are useful for tasks such as manual pipe inspection or plume tracking. The second type of vehicle group is the AUVs for long term—mainly autonomous—missions, like ECA A9 (http://www.ecagroup.com/en/solutions/a9-e-auv-autonomous-underwater-vehicle) or LAUV. These vehicles have only one main thruster which provides thrust for forward motion only. Directional control is done using multiple control surfaces called fins. Since the two kinds of vehicles are different in their control surfaces, FDI and control reallocation techniques are different as well.

Although the vehicle we use—BlueROV2—is a 6-DOF ROV, and the original Pixhawk + Raspberry Pi3 onboard computers provide only manual operation, using the simulator and ROS this vehicle is converted to an AV. Further research will be performed to transition the simulated vehicle and codebase onto the real BlueROV2.

### 4.2. Implementation

#### 4.2.1. Autonomy Manager

On the original BlueROV2 there is a Pixhawk 1 low-level controller, with ArduSub firmware acting as an attitude, depth sensor and actuator middleware [32]. The main controller, the so-called ‘companion computer’, is a Raspberry Pi3b using custom code with MavProxy which translates control inputs coming from the Raspberry Pi to Mavlink messages compatible with Pixhawk. BlueROV2 has 6 thrusters: 2 for *Z* axis vertical motion and roll stabilization and 4 for planar motion in the XY plane. The planar thrusters are aligned in a 45-degree setup. With this 6-DOF layout the vehicle can move in the longitudinal and lateral axis (forward and sideways) in manual mode. With the ‘UUV simulator’ DP controller it is considered to be a forward motion vehicle with heading control—like an AUV—without sideways motion. The 45-degree thruster layout provides redundancy for this kind of motion and allows thruster force to be reallocated to balance thrust and torque loss in the event of a thruster degradation. The minimal setup of the ArduSub is 2 parallel thrusters for longitudinal motion and heading control and one thruster for depth control. With the 6 thrusters of the BlueROV, it is redundant in hardware for every single partial or total thruster degradation, as well as some multiple partial thruster degradations.

The controller stack in the ‘UUV simulator’ uses internal waypoints (WP) to run the internal DP controller input. We create a command mode similar to the fly-by-wire-a (FBWA) mode in Ardupilot or the ‘Carrot’ chase type control in Paparazzi autopilot [33]. During every control update, our interface creates a temporary waypoint 50 m ahead of the vehicle with the specific parameters and the DP controller follows this point. The control movement is using a given Heading-Speed-Depth (HSD) command with a relative heading change, an absolute target speed and an absolute target depth command. Using this HSD from the different mission types like pipe following or mission waypoint following, the HSD is translated to this internal, temporary WP in every update. This makes the vehicle flexible to the mission types and gives a seamless path output using the correct DP controller parameters. It is robust to moderate environment noises like underwater disturbances thanks to the internal controller. If the heading change is greater than 90 degrees, the controller directs the UUV to make a three-point turn using reverse thrust, which allows steep heading changes, including complete heading reversal, to be made. In every mission the heading turn rate is limited typically to 30 deg/s.

**Figure 3:**
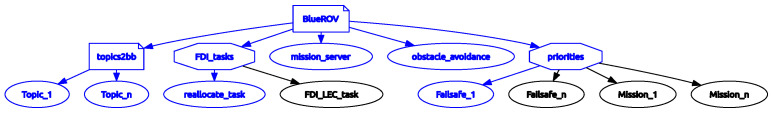
High-level BT in RQT.

The heart of the Autonomy Manager is a Behavior Tree-based control logic. The system consists of three groups of nodes from the Root node. The root itself is Parallel type, so all its children run concurrently without preemption or priority among them. Figure 3 presents the high-level view of the tree. The first group is a subtree of the blackboard update nodes. All of these are subscribed directly to a ROS topic of the BlueROV2 such as like battery information, distance from home or assurance monitor output. These nodes are reading and storing the value of the corresponding topic to a ROS complex type BB variable. This process is automatic in every tick.

In the update method for these nodes, the code performs comparison against preset threshold values. When a threshold is crossed, a specific variable is set to a predefined state. Typically, these variables are used as a warning flag, e.g., when the battery reaches a critical state the bb_battery_warning will be set to True, and other nodes can react to this flag. This subtree is always running each node in parallel, so no non-updating ROS message blocks another one.

The second group is a set of high-level parallel nodes, namely: FDI subtree, obstacle avoidance subtree and the mission server node. The FDI subtree runs the FDI LEC which is described in Section 4.3.1 LEC section. It also has a check_reallocation node. Each ‘check’ node makes a comparison of a given BB variable against a preset value, and the comparison result will be the state of the node (Success or Failure). Depending on this output, a sequential task can be run if the check fails using the ‘Success is Failure’ (SiF) BT node type.

The obstacle avoidance subtree creates the control output (HSD commands) from the tree. It is the last step in the control logic and can access all the mission related HSD commands. The code behind obstacle avoidance is using an obstacle map, which is a Grid ROS type. This grid map is updated using a low-cost distance sonar (EchoSounder) that gives a distance reading only, not detailed information compared to the imaging sonars. Based on the AUV actual (estimated) position and the distance reading, an obstacle shape is introduced to the map. In the Gazebo simulation, a uniform and scalable noise can be added to the raw sonar data to mimic the real and noisy reading of the ranging sonar. An additional LEC is created to filter out this noise using long short-term memory (LSTM) architecture.

The obstacle avoidance compares the AUV orientation and the mission heading with the obstacle map and checks for an obstacle-free heading to these directions. If there is any obstacle in the way of the AUV or mission, the avoidance kicks in and produces an appropriate HSD command. The avoidance heading is calculated based on the mission directives, e.g., in underwater infrastructure (pipe) following mode, the obstacle avoidance tries not to cross over the pipe to prevent pipe position estimation loss using the side scan sonar data.

The mission server is the mission controller. In the beginning, it reads the mission file—a YAML file which contains multiple mission descriptions. There are two main mission categories: pipe tracking with side scan sonar or waypoint mission with predefined waypoint, path list or a search pattern for search and rescue missions with automated localization of an underwater locator beacon (analogous to an aircraft flight data recorder—FDR). Each has its own set of parameters, target values and assurance directives with specific exit strategies. For example, if there is an obstacle detected closer than a safe margin, then it will abandon the mission and go to the surface while avoiding any obstacles. The server loads the missions sequentially and sets the actual mission type with its parameters in the BB. Once the mission completes, the next mission is loaded the same way until there are no missions remaining. At the end, it commands the AUV to return to home (RTH).

The third and most complex subtree runs the mission execution. It is a Selector type base node, called Priorities. In the mission execution, there are two base modes: pipe following and waypoint following, as described earlier. This subtree handles the mission level and the AUV level contingency actions as well. These contingency actions are the so-called ‘failsafes’, similar to the Ardupilot autopilot.

Available failsafes:

- BATTERY_LOW, when battery level is equal or less than AUV *failsafe battery low threshold*. Selected action: Surface AUV.
- SENSOR_FAILURE, when sensor failure occurs, e.g., RPM sensor. Selected action: Surface AUV
- OBSTACLE_STANDOFF, when the detected obstacle is closer than the given mission level threshold
- RTH, when battery level is at a boundary point compared to home distance, meaning it is the last chance to return to home with the actual battery level. Selected action: RTH if function is enabled
- GEOFENCE, when reached maximum distance from home. Selected action: RTH
- PIPE_LOST, when pipe lost more than *failsafe tracking lost threshold* seconds ago. This is 120 s by default, which is sufficiently long to avoid false-positives due to buried sections of pipe. If the pipe is lost during pipe tracking, the AUV enters loiter mode and will return to pipe tracking mode once the pipe is detected again.

Each failsafe is set in a hierarchy, in the order of the list above. In the subtree, they are at the same level with the mission nodes. Thus, the mission execution can be suspended if a failsafe occurs. Even a low-level failsafe like GEOFENCE can be suspended by the highest-level BATTERY_LOW failsafe. The Selector type ensures that only the highest priority child is running, either a failsafe of mission from the Priorities. Which one is active depends on the Checks (SiF type nodes). Each failsafe has one check based on comparing current values in the BB against preset thresholds. The active mission is set by the Mission Server, based on the mission execution state.

The mission subtree base nodes are Sequential BT nodes. Both have a Check for the requested mission type, thus they are in a Running state only when the actual mission is set by the mission server and there are no active failsafes. For the pipe tracking there is a specific task which generates the HSD output for the mission, and there are a.m. related nodes. One such a.m. node listens to the side scan sonar imaging LEC and checks whether its inputs are conformant with the LEC training data. The output of the a.m. provides an indicator whether the LEC output is useful or unreliable. If the a.m. output starts to rise, a leaf task in the BT is triggered and commands the AUV to proceed with minimal forward speed. If it is still not enough and the a.m. output rises even further, the tree disables pipe position estimation task via the pipe estimation enable/disable leave tasks. When the a.m. output returns to a nominal level, the tree switches back to the estimation task and commands full forward speed. The physical battery life constrains of the BlueROV and it does not allow for tracking the infrastructure as long as a torpedo shaped AUV (e.g., ECA A9) can. For this reason pipe tracking does not have any exit condition other than battery level, but it is easy to extend the tree for pipe length or maximum tracking time limits using the same logic for a long operation range AUV.

For the waypoint mission there are also checks for mission type and mission completion. If, for example, the waypoint mission type is ‘Search and Rescue for FDR’, when all the waypoints are completed from the search pattern an FDR location estimation task runs and creates a final waypoint for the estimated position. This waypoint is a Loiter_n_turns type which makes the AUV loiter in a circle around a given point on the seafloor with a given radius. During this, side scan sonars are active, and the waterfall image can be analyzed for debris and possible FDR signs.

#### 4.2.2. Mission Execution

A general mission specification contains following objectives: generate a desired path is given from the initial AUV position to the area of the target infrastructure with the minimum number of waypoints. These waypoints can be defined in the local coordinate system (North, East, Down—NED), global coordinate system (Latitude, Longitude, Altitude in decimal degree format—LLA) or with relative headings from the starting position. The next mission is the pipe following mode, which begins once the AUV is positioned in the operating area and has acquired the infrastructure using the side scan sonars. In this mode the AUV follows the pipe, including any bends and buried sections. If the pipe is lost, the PIPE_LOST failsafe activates, and the AUV starts to loiter looking for the pipe as described earlier. Other failsafe conditions can occur such as GEOFENCE, RTH or BATTERY_LOW. Each of these commands the AUV to the surface at the end. If there is enough battery charge left, the AUV returns to the home (initial) position before surfacing. During all these actions, the obstacle avoidance and degradation detection is active. The degradation management is the topic of the next chapters, where it is presented in detail.

### 4.3. LEC Assurance Monitoring and Decision Making

#### 4.3.1. Learning-Enabled Component for Fault Detection and Isolation

The objective of the fault detection component in the BlueROV2 is to observe and interpret the operation of the 6 thrusters, as well as the heading change commands, and to detect when a thruster reallocation is needed. This operation is handled by a Deep Neural Network (DNN), called FDI LEC, because of its ability to handle the uncertainty and variability of the real world. The input data for the FDI LEC is a 13-element vector containing the 6 thruster input commands, the 6 thruster RPMs and the heading change command. The thruster RPMs are a function of the commanded velocity and heading.

DNNs are generally non-transparent and reasoning about individual classifications is problematic, which makes their integration in safety-critical systems challenging. For this reason, the FDI LEC needs to be complemented with *assurance monitoring* and *assurance evaluation* functions to determine the correctness of each classification as shown in the Autonomy Manager subset in Figure 2. During the system operation of the BlueROV2, inputs regarding the thruster operation arrive one by one. After receiving each input the FDI LEC detects: (1) whether a thruster is faulty and (2) the level of degradation of the faulty thruster. The assurance monitor then computes a credibility and confidence value for the FDI LEC’s classification. The assurance evaluator combines these two values to decide whether the classification is trustworthy.

Because of the low-dimensional input space, the FDI LEC is chosen to be a fully connected, feed-forward DNN with 3 hidden layers. Details regarding the architecture are presented in Table 1. This architecture is chosen to achieve good detection accuracy for the task, avoid over-fitting, and keep computational requirements low while also explore how a general-purpose and easily implementable DNN can be integrated in an assurance monitoring and evaluation framework.

#### 4.3.2. Assurance Monitoring

The role of the a.m. is to quantify how trustworthy the output of the LEC is. The Inductive Conformal Prediction (ICP) framework [34] allows computing credibility and confidence values for a particular LEC classification. We consider a training set {$z_{1},\ldots ,z_{l}$} of examples, where each $z_{i}\in Z$ is a pair $\left(x_{i},y_{i}\right)$ with $x_{i}$ the feature vector and $y_{i}$ the label of that example. Central to the application of ICP is a *nonconformity function* or nonconformity measure (NCM) which quantifies how different a labeled input is from the examples in the training set. For a given test example $x_{i}$ with candidate label ${\tilde{y}}_{i}$, a NCM computes an indicative value of how strange the pair $\left(x_{i},y_{i}\right)$ is, given the training set $\left\{z_{1},\ldots ,z_{i-1},z_{i+1},\ldots ,z_{n}\right\}$. There are many proposed NCMs that can be used [35,36,37,38,39]. In this work, we chose the nearest centroid NCM for its performance in real time applications.

When two input vectors $x_{1}$ and $x_{2}$ are similar, they are expected to have the same labels. To compute the similarity between any two input vectors we use a Siamese network [40]. A Siamese network $f_{d}$ maps an input vector *x* into an embedding representation $v=f_{d}\left(x\right)$ and is trained to minimize the Euclidean distance between the embedding representations of inputs belonging to the same class while maximizing it for inputs belonging to different classes. Since the representations of data points with the exact same label are expected to be close to each other in the embedding space, for each class $y_{i}$ we compute its centroid $\mu_{y_{i}}=\frac{\sum_{j=1}^{n_{i}}v_{j}^{i}}{n_{i}}$, where $v_{j}^{i}$ is the embedding representation of the *j*th training example from class $y_{i}$ and $n_{i}$ is the number of training examples in class $y_{i}$. The NC function is then defined as

(1) $$\alpha \left(x,y\right)=\frac{d(\mu_{y},v)}{\min_{i=1,\ldots ,n:y_{i}\neq y}d\left(\mu_{y_{i}},v\right)}$$

where $v=f_{d}\left(x\right)$. It should be noted that for computing the nearest centroid NCM, we need to store only the centroid for each class regarding a training set, no matter how large the training set may be.

The NCM a(x,y) is a measure of dissimilarity between a test input *x* with candidate label *y* and the training data $z_{1}\ldots z_{l}$. When the Euclidean distance between $v=f_{d}\left(x\right)$ and the centroid assigned to the class *y* is small, this is an indication that the DNN has been trained in similar examples while a large distance value indicates an uncommon example. Hypothesis testing is used to mathematically define what small and large are in this context. The training set $z_{1}\ldots z_{l}$ is split into two parts: (1) the proper training set $z_{1}\ldots z_{m}$ of size m<l that is also used for training the Siamese network and the FDI LEC, and (2) the calibration set $z_{m+1}\ldots z_{l}$ of size l−m. We first compute the NCMs $a(x_{i},y_{i})$, i=m+1,…,l for the examples in the calibration set. Given a test example *x* with an unknown label *y*, for each candidate label ${\tilde{y}}_{j}$, j=1,…,c, ICP computes a *p*-value $p_{j}$ as the fraction of the calibration nonconformity scores that are equal or larger than the nonconformity score of the test input $\alpha (x,{\tilde{y}}_{j})$:

(2) $$p_{j}\left(x\right)=\frac{\left|\{\alpha \in A:\alpha \geq \alpha (x,j)\}\right|}{\left|A\right|}$$

These *p*-values are then used to compute the classification *credibility* and *confidence*. Assuming the FDI LEC produces a classification $\hat{y}$:

(3) $$\mathrm{credibility}=p_{\hat{y}}$$

(4) $$\mathrm{confidence}=1-\max_{j=1,\ldots ,c:j\neq \hat{y}}p_{j}$$

The credibility shows how credited $\hat{y}$ is and the confidence shows how special it is compared to the other possible labels. These two metrics define the following four scenarios (Table 2):

#### 4.3.3. Assurance Evaluator

The *p*-values computed by ICP are indicative of which of the FDI LEC predicted labels are trustworthy. The quality of the predictions can be evaluated through the credibility and confidence metrics. As presented in Table 2 trustworthy classifications tend to have high *p*-values and higher than the *p*-values of the rest of the classes. The assurance evaluator decides if a classification can be trusted and if not will raise an alarm which may require further investigation. For this operation we use the concept of *selective classification* [41,42]. A selective classifier (f,g) decides whether to keep the classification from an underlying model or reject it and is defined as:

(5) $$\left(f,g\right)\left(x\right)≜\{\begin{array}{cc} f(x), & \mathrm{if}\;g(x)=1\\ \mathrm{reject}, & \mathrm{if}\;g(x)=0 \end{array}$$

where *f* is a classifier, and g:X→{0,1} is a selective function that we call *assurance evaluator*. Consider a function *k* that evaluates the classifications of *f* and a threshold θ. The selective function *g* is defined as, $g_{\theta }\left(x|k,f\right)=1\left[k\left(x,{\hat{y}}_{f}\left(x\right)|f\right)>\theta \right]$. A selective classifier is evaluated using the *coverage* and *risk* metrics. Coverage is a metric of the frequency *f* classifications are accepted by *g*. Risk is the error-rate in the accepted classifications. These measures can be empirically evaluated using any finite labeled set $S_{m}$. The empirical coverage $\hat{\phi }$ and risk $\hat{r}$ are computed as:

(6) $$\begin{array}{cc} \hat{\phi }\left(f,g|S_{m}\right) & ≜\frac{1}{m}\sum_{i=1}^{m}g\left(x_{i}\right) \end{array}$$

(7) $$\begin{array}{cc} \hat{r}\left(f,g|S_{m}\right) & ≜\frac{\frac{1}{m}\sum_{i=1}^{m}l\left(f\left(x_{i}\right),y_{i}\right)g\left(x_{i}\right)}{\hat{\phi }\left(f,g|S_{m}\right)} \end{array}$$

where $l(f\left(x_{i}\right),y_{i})=1$ if $f\left(x_{i}\right)=y_{i}$ otherwise $l(f\left(x_{i}\right),y_{i})=0$.

For a given classifier *f* we optimize the assurance evaluator *g* based on the area under the RC curve (AURC) defined in [43]. Consider an independent set of *n* labeled points $V_{n}$ and let the set $\Theta ≜\{k\left(x,{\hat{y}}_{f}\left(x\right)|f\right):\left(x,y\right)\in V_{n}$}. Using every value in Θ as a threshold for *g* we can compute *n* empirical risk and coverage values and plot a risk-coverage (RC) curve. The choice of *k* affects the performance of the assurance evaluator and it is preferable for the rejected points to be otherwise incorrectly classified so that we may have high coverage with low risk. The AURC is used to evaluate the performance of a pair (f,g),

(8) $$\mathrm{AURC}\left(k,f|V_{n}\right)=\frac{1}{n}\sum_{\theta \in \Theta }\hat{r}\left(f,g_{\theta }|V_{n}\right).$$

A function *k* needs to be chosen to minimize AURC, which intuitively minimizes the average empirical risk of a given empirical coverage value.

The assurance evaluator is constructed with a choice of a classification evaluator function *k* and a threshold θ. *k* is expressed as a linear combination of the credibility and confidence, computed by the assurance monitor,

(9) $$k\left(x,{\hat{y}}_{f}\left(x\right)\right)=a*\mathrm{credibility}\left(x,{\hat{y}}_{f}\left(x\right)\right)+b*\mathrm{confidence}\left(x,{\hat{y}}_{f}\left(x\right)\right).$$

The computation of the pair of values (a,b) that lead to the optimal assurance evaluator happens during design time using an independent labeled set $V_{n}$ that has not been used for training *f*. We perform a grid search for a,b∈[0,1] that minimize the AURC and yields the optimal risk and coverage curve. Based on the RC curve and the mission requirements regarding the accepted risk and coverage of the assurance evaluator $\left(r^{*},c^{*}\right)$, a threshold θ is chosen such that $\left(\hat{r},\hat{c}\right)=\left(r^{*},c^{*}\right)$.

### 4.4. Decision Making

The output of the FDI LEC is a class which represents either a nominal state or identifies the faulty thruster, with the approximate level of degradation. The representation is non-linear. There are 22 classes, one (#21) for nominal. For the *Z* axis thrusters (no and 5) there is only one degraded class (#20). The AUV considered nominal, if the thruster efficiency is greater than or equal to 90%, and faulty otherwise. There is no reallocation for these two, since we cannot balance the torque with the other 4 for depth control and balancing 4 or 5 to the same level might cause the AUV to be stuck underwater without the necessary thrust to surface. For *Z* axis thruster degradation the system only raises an alarm through the BB nodes. If these warnings persist the AUV goes to the surface.

For the planar thrusters, we distinguish two levels of degradation: severe if the efficiency is between 0 to 50%, and mild degradation if it is between 50 to 90%. Over 90% it is classified as nominal. There is one severe class covering the lower 50% efficiency and four mild classes with 10% efficiency steps for each thruster. For the reallocation of the mild cases, the class center is used. For example, if the ground truth efficiency was 63% it is classified to the 60–70 range, and the reallocation is done for 65%. Thus, if the classification is correct, the thrust error after reallocation will be a maximum of 5%, which will not cause major heading offset error.

The mild degradation means the thruster has some efficiency loss, e.g., partially blocked blades due to seaweed or other object, but remains operational and safe for the AUV to use it further. For this degradation, we must balance the torque loss with the opposing side thruster through the Thruster Allocation Matrix (TAM) provided by the ‘UUV simulator’. It describes the relation of the 6-DOF motion of the AUV with its custom thrusters. The operation is done in the reallocation leaf of the FDI subtree of the BT.

Severe degradation means it has some serious issue (e.g., fishing net caught in thruster, broken blade(s), damaged motor or speed controller electronics). In this case, it is unsafe to continue using that thruster. To prevent further damage and lower the risk, the control reallocation leaf turns off the degraded thruster and its pair. Thus, the torque will be in balance, but the sum of all thrust will be half of the original for planar motion. The AUV speed will be considerably lower compared to the nominal state, but it is still operational and can continue the mission.

### 4.5. Assurance Monitor Design and Execution

To present in detail the steps needed to design the assurance monitor and evaluator as well as the system execution, Algorithms 1 and 2 summarize the offline and online algorithms.

At design time, the assurance monitor is calibrated to quantify the trustworthiness of each classification. The idea is that an LEC will likely classify an input correctly when its training dataset containing similar input data. First, the LEC classifier and the Siamese network used for the assurance monitor are trained with the proper training set. Calibration of the assurance monitor based on ICP is achieved by computing the NC scores of the calibration data using the nearest centroid NCM. These NC scores are stored and used to compute the credibility and confidence values for each classification. The design of the assurance evaluator performed offline using test data and aims at computing the evaluator function that minimizes the AURC as well as choosing a threshold for the selective function. The threshold is chosen according to the application requirements regarding the acceptable decision frequency and error-rate by choosing an operation point on the RC curve.

During execution, when the system receives an input, the LEC classifier generates a classification. The Siamese network first transforms the input to an embedding classification, and the assurance monitor computes a *p*-value for each possible class by comparing each class’s NC score with the ones computed on the calibration set during design time. Then, the credibility and confidence values are computed for the particular classification. The assurance evaluator computes a linear combination of the credibility and confidence and decides if the classification can be trusted or not based on the chosen threshold.

**Algorithm 1** Design time**Input:** proper training data (X,Y), calibration data $\left(X^{c},Y^{c}\right)$, offline test data $\left(X^{t},Y^{t}\right)$.1: Train the classification LEC *f* with (X,Y) as training set and $\left(X^{c},Y^{c}\right)$ as validation set.
2: Train the Siamese network $f_{d}$ with (X,Y) as training set and $\left(X^{c},Y^{c}\right)$ as validation set.3: // Compute the nonconformity scores for $\left(X^{c},Y^{c}\right)$ using Equation (1).4: $A=\{\alpha \left(x,y\right):\left(x,y\right)\in \left(X^{c},Y^{c}\right)\}$.5: Compute the *p*-values for all the classes of the data in $\left(X^{t},Y^{t}\right)$ using Equation (2).6: Compute the credibility and confidence for the data in $\left(X^{t},Y^{t}\right)$ using Equations (3) and (4).7: Perform a grid search to compute the coefficients a,b to define the evaluator function *k* shown in Equation (9) to minimize the AURC shown in Equation (8).8: Construct the set $\Theta =\{k\left(x,{\hat{y}}_{f}\left(x\right)|f\right):\left(x,y\right)\in \left(X^{t},Y^{t}\right)\}$.9: Using every value in Θ as a threshold for the selective function *g*, plot the Risk-Coverage curve according to Equations (6) and (7). This is used to select an operation point (threshold) θ according to the application requirements.

**Algorithm 2** Execution time**Input:** Classification LEC *f*, Siamese network $f_{d}$, nonconformity scores *A*, evaluator function *k*, threshold θ for the selective function *g*, test input $x_{t}$.1: Compute the classification ${\hat{y}}_{t}=f\left(x_{t}\right)$.2: Compute the embedding representation $v_{t}=f_{d}\left(x_{t}\right)$.3: **for** each possible class *j*
**do**4:     Compute the nonconformity score $\alpha (x_{t},j)$ using Equation 1.5:     $p_{j}\left(x_{t}\right)=\frac{\left|\{\alpha \in A:\alpha \geq \alpha \left(x_{t},j\right)\}\right|}{\left|A\right|}$.6: **end for**7: Compute the credibility and confidence for $\left(x_{t},{\hat{y}}_{t}\right)$ according to Equations 3 and 4.8: **if**
$g_{\theta }\left(x|k,f\right)=1$
**then**9:     **return**
${\hat{y}}_{t}$.10: **else**11:     **return** No decision.12: **end if**

### 4.6. Decision-Making Execution-AUV Example

Section 4.5 presents the architecture and the algorithm for the AM. In this section, we illustrate the algorithm using an AUV example. Algorithm 3 below describes in detail how the method is applied for decision-making in the BlueROV fault-adaptive autonomy example. Once the control logic receives the assured output from the LEC AM, based on the output class and the BlueROV2 vehicle specific parameters, it preforms the control reconfiguration as necessary.
**Algorithm 3** Execution-time Steps for BlueROV example**Input:** Classification LEC *f*, siamese network $f_{d}$, nonconformity scores *A*, evaluator function *k*, threshold θ for the selective function *g*, real-time input $x_{t}$.1: Compute the classification ${\hat{y}}_{t}=f\left(x_{t}\right)$.2: Compute the embedding representation $v_{t}=f_{d}\left(x_{t}\right)$.3: **for** each possible class *j* **do**4:     Compute the nonconformity score $\alpha (x_{t},j)$ using Equation (1).5:     $p_{j}\left(x_{t}\right)=\frac{\left|\{\alpha \in A:\alpha \geq \alpha \left(x_{t},j\right)\}\right|}{\left|A\right|}$.6: **end for**7: Compute the credibility and confidence for $\left(x_{t},{\hat{y}}_{t}\right)$ according to Equations (3) and (4).8: **if**
$g_{\theta }\left(x|k,f\right)=1$
**then**9:     Get degraded thruster id ID and efficiency η from class ${\hat{y}}_{t}$10:     **if** η>90% **then**11:         **return** Nominal State.12:     **else**13:         **if** ID is in [4,5] **then**14:            Show *‘Z axis degradation warning’*15:            **return** Degraded State - no control reconfiguration required16:         **else**17:            **if** η<50% **then**18:                Show *‘Severe XY axis degradation warning’*19:                Get ID thruster pair $ID_{p}$ from definition20:                Turn off ID and $ID_{p}$21:            **else**22:                Show *‘Mild XY axis degradation warning’*23:                Get ID thruster pair $ID_{p}$ from definition24:                Perform control reallocaton - set $ID_{p}$ to η to balance torque loss25:            **end if**26:            **return** Degraded State - control reconfiguration complete27:         **end if**28:     **end if**29: **else**30:     **return** Nominal State - LEC output not trustworthy.31: **end if**


## 5. Results

For data gathering, multiple waypoint-following missions were created, including planar and vertical axis thruster degradations. There was a total of 349 experiments simulated. Each experiment was 300 s long with 1Hz resolution. The initial 20 s of each simulation was considered invalid since the ROS nodes are booting up, the output of several nodes are incorrect during this time. This leaved an effective time of 280 s for each simulation and covered the nominal and all the degraded classes with efficiencies from 0% to 100%. Labeled data points gathered from the simulations were then shuffled and split to form the proper training set, with 80% of the available data, and the calibration set with the remaining 20%.

The proper training set was used to train the FDI LEC as well as train the Siamese network, part of the assurance monitor. The calibration set was used to compute the calibration NC scores as shown in (1) and was also used as validation set during the training of the FDI LEC and the Siamese network. Both the FDI LEC and the Siamese network had the same architecture with only difference that the Siamese network lacked the output SoftMax layer with 22 units. Because of the relatively small size and 1-D shape of the input, we used a fully connected DNN with three hidden layers and ReLU activation functions. The first had 256 units, the second had 32 and the last had 16 units. In the case of the Siamese network, the last hidden layer computed the embedding representations. The training and validation accuracy of the FDI LEC were 0.925 and 0.926, respectively. We evaluated the performance of the Siamese network in clustering data belonging in the same class using the silhouette analysis [44]. The silhouette value s(i)∈[−1,1] of a data point *i* showed how close it was to data points of the same class and far from data points of different classes. The mean s(i) over all data of the entire dataset was a measure of how appropriately the data had been clustered. The mean silhouette value of the training set and the calibration/validation set were both 0.64 showing that the embedding representations computed using the Siamese network were placed in clusters according to their similarity.

**Figure 4:**
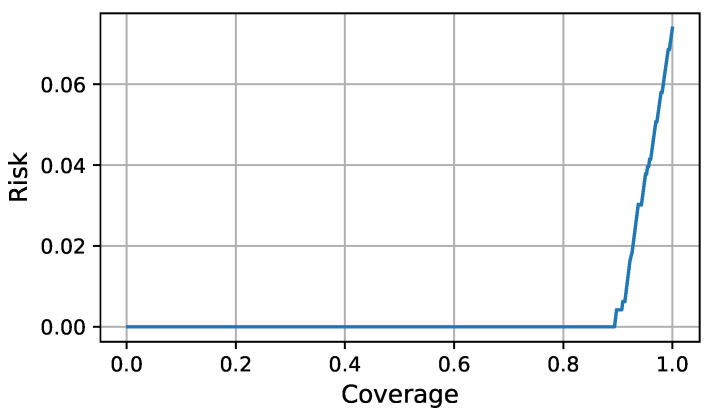
Optimal Risk-Coverage curve.

During execution time the FDI LEC is complemented with the assurance monitor and the assurance evaluator to decide whether a classification could be trusted. For a given test input and FDI classification, the assurance monitor computed the credibility and confidence through Equations (3) and (4). The assurance evaluator decided about the quality of the classification by comparing the classification evaluation function value (9) with a chosen threshold. To compute the optimal classification evaluator function as a linear combination of the credibility and confidence during design time, we collected eight test sequences, representative of the simulated missions. We performed a grid search for a,b∈[−1,1] to find the linear combination of credibility and confidence that minimized the AURC using the offline test data. The optimal Risk-Coverage curve is shown in Figure 4 and was generated using a=0.9 and b=−0.3. The leftmost point in the curve was achieved when no decision is taken because the chosen threshold could not be reached by the classification evaluation function. The rightmost point in the curve was achieved when a decision was taken for every classification. An operation point could be chosen according to the requirements for accuracy and decision frequency.

A total of 60 waypoint scenarios were made with different random seeds, with and without control reallocation, for each fault class with possible control reallocation (classes #0 to #19, without #20 for z axis thrusters and nominal #21). These scenarios were similar to the training and test data missions, but with a fixed number (five) of waypoints. The scenario was made with and without FDI LEC and control reallocation to compare the results.

These simulations were similar to the training dataset, but had intense heading changes, resulting in overall lower accuracy compared to the training/validation/test data. The reason for this was to check the a.m. technology how well can it preform in hazardous environment—close to obstacles. Table 3 presents the metrics with different assurance technologies. Applied threshold is the value used for the corresponding technology. For real-time operation, the goal was an overall high recall and accuracy value—accepting all correct LEC outputs while rejecting incorrect ones.

**Figure 5:**
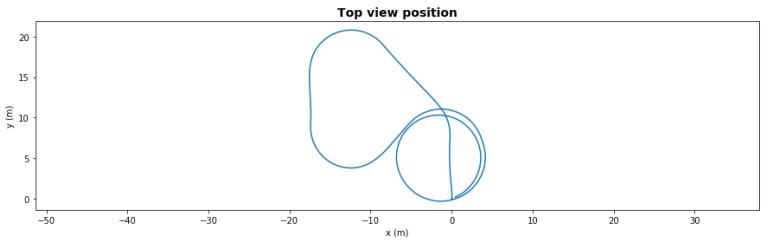
Waypoint mission—nominal path.

Figure 5 presents a five-waypoint mission scenario. The total simulation time was 150 s. The AUV started from 0,0 coordinates and followed the generated waypoints. After completing the last waypoint, the AUV turned into loiter mode.

In a nominal state, the AUV completed the mission in an average of 88.67 s with *1.69* m cross-track error.

**Figure 6:**
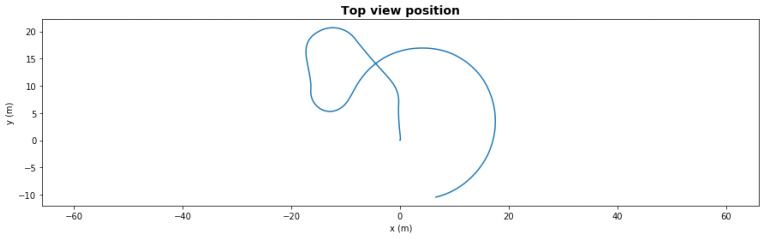
Waypoint mission—path with severe degradation.

**Figure 7:**
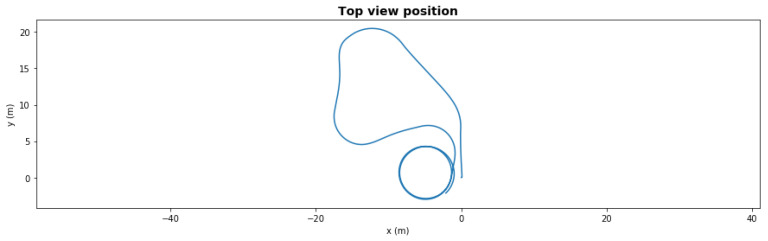
Waypoint mission—path with severe degradation and reallocation.

Figure 6 presents the same scenario with a severe degradation, the front left thruster had efficiency dropped to 41% at t = 50 s during the second left turn. With this severe degradation, the AUV could not take the last right turn towards the waypoint since it lacked the necessary torque. With the fault-adaptive autonomy in the same setup, the FDI LEC classified the degradation correctly 1 s after it occurred, and the system made a control reallocation decision—turning off the front severely degraded thrusters. The AUV still finished the path, shown in Figure 7, but the final loitering trajectory was different due to loss of front thrusters.

**Figure 8:**
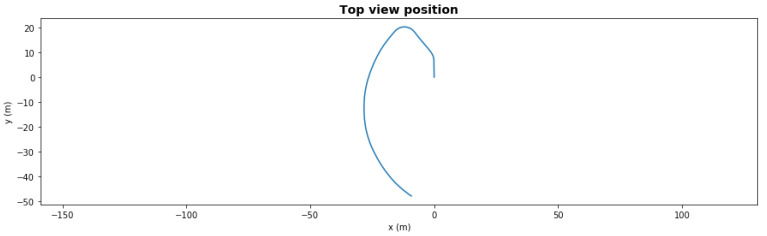
Waypoint mission—path with mild degradation.

**Figure 9:**
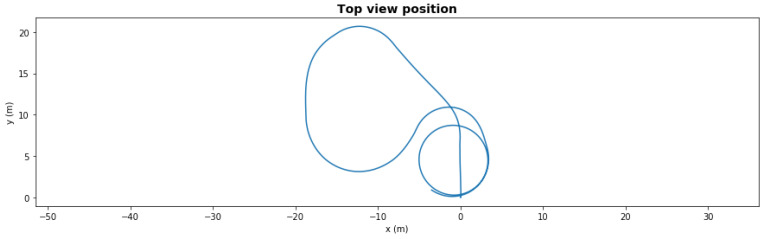
Waypoint mission—path with mild degradation and reallocation.

Figure 8 and Figure 9 show the same scenario with mild degradation (rear right thruster had a degradation with 66.5% efficiency at t = 50 s). The FDI LEC decided in under 3 s and reallocated the rear left thruster to 65%. Without reallocation, the AUV could not finish the mission, but with reallocation it successfully completed in under 150 s.

In Table 4, simulation results without the FDI LEC shows the intense effect of degradation to the mission. In 27 out of 60 cases, the AUV could not finish the mission due to loss of control. A value of ‘*−1*’ in the in *Time to complete* column indicates all simulations failed to complete the mission in that class. The average cross-track error was *5.75* m and the average time to complete the mission was *90.55* s.

In Table 5, the *averaged* simulation results can be seen—the mission completion time in seconds with and without FDI. Without FDI, the AUV could not finish the mission in 6 out of 60 cases due to the degradation. With FDI, the AUV finished more cases with a shorter average completion time (*88.21* s vs. *90.55* s) under the 150 s simulation time limit. In general, based on these simulations, the BlueROV was more sensitive to rear thruster degradations. Even with the FDI, in some cases it still could not make the mission in time as classes #10 and #15 indicate with values of ‘*−1*’ in column *Time to complete with reallocation*. The average time for reallocation was *2.42* s, which means the FDI successfully detected the problem from 2–3 samples after introducing degradation to the system. The average cross-track error during simulation was *1.63* m.

Finally, In Table 6, the average metrics of the mission execution time and the cross-track error are shown. As expected, the AUV in degraded state performed worse (2.6% slower and with 3.5 times higher cross-track error), while the AUV with fault-adaptive autonomy performed very close to the nominal state.

## 6. Discussion

We have presented a fault-adaptive system architecture for Learning-Enabled Component (LEC) equipped autonomous vehicles operating in a hazardous environment. We have implemented a Deep Neural Network (DNN) called Fault Detection and Isolation (FDI) LEC with an Inductive Conformal Prediction (ICP) framework-based Assurance Monitor (AM). With the a.m. in the Cyber-Physical System (CPS), we quantify how trustworthy the LEC output is in real time. The Autonomy Manager captures the information provided by the FDI LEC and commands a control reallocation if necessary to maintain control and complete mission objectives. The autonomy system applied to an Autonomous Underwater Vehicle (AUV) based on BlueROV2 has several contingency actions in the vehicle and in the mission level such as battery low failsafe or pipe lost failsafe which all triggers a safety action.

The real-time assurance monitor for the LEC in the CPS provides a secure and reliable operation for the FDI system. The new a.m. Assurance Evaluator (AE) can significantly raise the overall performance of the LEC output (raw LEC recall is 84.05% vs. LEC with a.m. and AE recall is 98.37%). With this high performance, the fault classification is dependable for a control reallocation in the middle of an autonomous mission—without a high risk of a possible incorrect fault isolation. Our fault-adaptive system architecture makes the AUV robust against hazards during operation in an unknown underwater environment.

Future extensions are planned for the BlueROV2 AUV to further raise the contingency possibilities and widen the safety of the autonomous systems.

## Figures and Tables

**Table 1:** FDI LEC architecture.

#	Layer	Layer Parameters
1	Input	13 units
2	Dense	256 units, ReLU
3	Dense	32 units, ReLU
4	Dense	16 units, ReLU
5	Output	22 units

**Table 2:** Scenarios that can be observed for different values of confidence and credibility.

Credibility	Confidence	Description
High	High	The preferred situation that usually leads into accepting the FDI LEC classification. $p_{{\hat{y}}_{j}}$ is high and much higher than the *p*-values of the other classes.
High	Low	$p_{{\hat{y}}_{j}}$ is high but there are other high *p*-values so choosing a single credible class may not be possible.
Low	High	None of the *p*-values are high for a credible decision.
Low	Low	A label different than ${\hat{y}}_{j}$ could be more credible.

**Table 3:** Metrics with different assurance technologies.

	LEC + a.m. + AE	LEC + AM, Credibility Threshold	Raw LEC, SoftMax Threshold	Raw LEC, NO AM
Applied Threshold	−0.1	0.6	0.99	-
Recall	98.37%	91.64%	21.45%	84.05%
Accuracy	93.85%	92.37%	33.24%	84.05%
Rejected	12.54%	29.50%	81.24%	0.00%

**Table 4:** Waypoint scenario without reallocation.

GT Degradation Thruster ID	GT Degradation Efficiency (%)	GT LEC Class	Cross Track Error (m)	Time to Complete (s)
0	41	0	5.54	*−1.00*
0	56.5	1	2.05	93.00
0	66.5	2	1.85	90.33
0	76.5	3	1.74	89.33
0	86.5	4	1.75	90.67
1	41	5	5.37	86.00
1	56.5	6	1.91	93.67
1	66.5	7	1.81	91.33
1	76.5	8	1.53	88.00
1	86.5	9	1.60	88.00
2	41	10	11.94	*−1.00*
2	56.5	11	16.27	*−1.00*
2	66.5	12	12.81	*−1.00*
2	76.5	13	3.00	88.50
2	86.5	14	1.74	91.00
3	41	15	12.94	*−1.00*
3	56.5	16	13.74	*−1.00*
3	66.5	17	9.74	*−1.00*
3	76.5	18	5.62	*−1.00*
3	86.5	19	2.10	93.00

**Table 5:** Waypoint scenario with reallocation.

GT Thruster ID	GT Efficiency (%)	FDI LEC Thruster ID	FDI LEC class	Cross Track Error (m)	Time to Complete (s)	Reallo- Cation Time (s)
0	41	0	0	1.37	87.00	1.41
0	56.5	0	1	1.63	87.00	2.42
0	66.5	0	2	1.53	85.00	1.38
0	76.5	0	3	1.68	88.33	2.12
0	86.5	0	4	1.78	92.00	4.44
1	41	1	5	1.36	86.00	1.41
1	56.5	1	6	1.60	87.33	1.36
1	66.5	1	7	1.64	88.50	1.40
1	76.5	1	8	1.81	91.00	2.37
1	86.5	1	9	1.66	88.00	2.04
2	41	2	10	5.73	*−1.00*	1.36
2	56.5	2	11	1.64	88.00	1.71
2	66.5	2	12	1.44	86.00	3.39
2	76.5	2	13	1.61	88.00	2.02
2	86.5	2	14	1.79	89.67	8.37
3	41	3	15	4.62	*−1.00*	2.05
3	56.5	3	16	1.90	91.00	2.40
3	66.5	3	17	1.75	90.33	1.71
3	76.5	3	18	1.63	88.67	1.36
3	86.5	3	19	1.60	86.00	3.37

**Table 6:** Waypoint scenario metrics.

	Time to Complete (s)	Cross-Track Error (m)
Nominal	88.21	1.63
Degraded	90.55	5.75
Degraded with FDI	88.66	1.69

## Data Availability

Not applicable.
